# Differential diagnosis checklists reduce diagnostic error differentially: A randomised experiment

**DOI:** 10.1111/medu.14596

**Published:** 2021-08-18

**Authors:** Juliane E. Kämmer, Stefan K. Schauber, Stefanie C. Hautz, Fabian Stroben, Wolf E. Hautz

**Affiliations:** ^1^ Department of Emergency Medicine, Inselspital University Hospital University of Bern Bern Switzerland; ^2^ Center for Adaptive Rationality (ARC) Max Planck Institute for Human Development Berlin Germany; ^3^ Centre for Health Sciences Education, Faculty of Medicine University of Oslo Oslo Norway; ^4^ Department of Anesthesiology and Operative Intensive Care Medicine (CBF), Charité – Universitätsmedizin Berlin Humboldt University of Berlin Berlin Germany

## Abstract

**Introduction:**

Wrong and missed diagnoses contribute substantially to medical error. Can a prompt to generate alternative diagnoses (prompt) or a differential diagnosis checklist (DDXC) increase diagnostic accuracy? How do these interventions affect the diagnostic process and self‐monitoring?

**Methods:**

Advanced medical students (*N* = 90) were randomly assigned to one of four conditions to complete six computer‐based patient cases: group 1 (prompt) was instructed to write down all diagnoses they considered while acquiring diagnostic test results and to finally rank them. Groups 2 and 3 received the same instruction plus a list of 17 differential diagnoses for the chief complaint of the patient. For half of the cases, the DDXC contained the correct diagnosis (DDXC+), and for the other half, it did not (DDXC−; counterbalanced). Group 4 (control) was only instructed to indicate their final diagnosis. Mixed‐effects models were used to analyse results.

**Results:**

Students using a DDXC that contained the correct diagnosis had better diagnostic accuracy, mean (standard deviation), 0.75 (0.44), compared to controls without a checklist, 0.49 (0.50), *P* < 0.001, but those using a DDXC that did not contain the correct diagnosis did slightly worse, 0.43 (0.50), *P* = 0.602. The number and relevance of diagnostic tests acquired were not affected by condition, nor was self‐monitoring. However, participants spent more time on a case in the DDXC−, 4:20 min (2:36), *P* ≤ 0.001, and DDXC+ condition, 3:52 min (2:09), than in the control condition, 2:59 min (1:44), *P* ≤ 0.001.

**Discussion:**

Being provided a list of possible diagnoses improves diagnostic accuracy compared with a prompt to create a differential diagnosis list, if the provided list contains the correct diagnosis. However, being provided a diagnosis list without the correct diagnosis did not improve and might have slightly reduced diagnostic accuracy. Interventions neither affected information gathering nor self‐monitoring.

## Abstract 2

The authors demonstrate that a differential diagnosis checklist reduces diagnostic error without influencing the extent and relevance of information gathering, but only if the correct diagnosis is contained in the checklist.

## 1 INTRODUCTION

Teaching students to diagnose a patient's condition is a key objective of medical education.^1^ But besides the skill's prominent role in most medical curricula, diagnostic errors constitute a major source of medical errors, which may lead to patient harm and even death.^2^, ^3^ Diagnostic errors, especially missed diagnoses, frequently result from a failure to consider the correct diagnosis or settling on a wrong diagnosis too early.^4^, ^5^ Hence, considering alternative diagnostic hypotheses may improve diagnosis, especially early in the diagnostic process.^6^, ^7^, ^8^ This is because the set of diagnostic hypotheses considered determines what diagnostic information physicians acquire (and omit) and how they interpret and integrate it.^9^, ^10^ For example, it has been shown that generating multiple hypotheses early on results in asking the patient more questions during a general practitioner (GP) consultation^11^ and leads to more complete and less biased documentation among GPs.^12^ Similarly, reflective practice, that is, a critical consideration of one's reasoning and decisions, may ultimately lead to fewer diagnostic errors, at least when facing difficult cases.^13^ In contrast, diagnoses that are not considered or considered only late in the process are less likely to be detected, even with incoming supporting information.^10^

These insights underlie efforts to reduce diagnostic errors by stimulating on‐site hypothesis generation among diagnosticians, for example, with the help of diagnostic checklists.^14^, ^15^, ^16^ Checklists may reduce the cognitive load of physicians of recalling all steps of a workup or of important differential diagnoses. This may be especially helpful during residency training, when junior physicians need their cognitive resources to train skills not covered in undergraduate education, such as management reasoning.^17^ Although widely used in other high‐risk settings such as operating rooms^18^, ^19^, ^20^ and despite their face validity, empirical evidence on the effectiveness of diagnostic checklists is scarce and contradictory.^21^, ^22^, ^23^, ^24^, ^25^, ^26^, ^27^ Moreover, little is known about their functioning and thus about how best to design them. We addressed this gap with an experimental study in an emergency room setting.

Different types of checklists have been proposed for their applicability during the diagnostic process.^14^ General checklists are symptom independent and provide a list of relevant steps to reach a diagnosis, such as to consider alternative diagnoses or to pause to reflect.^14^, ^16^, ^28^ Such checklists are intended to help reduce diagnostic errors as they may enforce the completion of critical steps during diagnosis or trigger deliberate thinking to circumvent cognitive biases.^29^ Although general checklists vary in the number and type of steps they list, they commonly entail the instructions to consider alternative diagnoses and their respective likelihood. But despite their intuitive appeal as economical and situation independent, research has not yet demonstrated substantial error reduction with their use,^21^, ^30^, ^31^ similar to other methods of general debiasing.^32^, ^33^ Here, we test whether the generic prompts to consider alternative hypotheses and rank them results in more accurate diagnoses.

Symptom‐ or disease‐specific checklists such as differential diagnosis checklists (DDXCs), in contrast, propose comprehensive lists of causes for specific presenting complaints.^14^, ^34^ A DDXC may act as a retrieval aid or reminder of frequent or commonly missed diagnoses and trigger consideration of more alternative diagnoses and hence further data gathering.^34^ Indeed, DDXCs have been shown to increase diagnostic accuracy in difficult cases.^21^ Yet tests on the robustness of this effect are missing. Also, given that it is hardly feasible to list all possible differential diagnoses in practice, (uncommon) diagnoses missing on a checklist may be considered even less. This hypothesis rests on the findings of related research on availability bias, showing that the presentation of a diagnosis may (falsely) prime diagnosticians in their subsequent diagnosis.^35^, ^36^ Also, there is evidence showing that, whereas computer aids providing correct advice may improve internists' ECG interpretation, they may lower accuracy when providing an incorrect interpretation.^37^ It is therefore of great practical importance to understand whether the inclusion of the correct diagnosis on a DDXC is crucial to its beneficial effect and whether its absence may even decrease the likelihood of making the correct diagnosis.

Moreover, it is not yet well understood how the utilisation of prompts or checklists impacts the diagnostic process.^38^ In fact, it is conceivable that enlarging the hypothesis space may lead to ‘excessive consultation or needless testing’ (p. 311)^14^ rather than to just remedy undertesting.^39^ Furthermore, influencing the way diagnosticians approach a problem may affect their meta‐cognitive monitoring, because the actual task changes from a having to create a diagnosis to merely having to select it (at least, when it is on the DDXC). Previous research on create versus select response tasks in assessments suggests that they differ in important characteristics of meta‐cognition.^40^

Here, we examine how the specific interventions discussed above affect (a) diagnostic accuracy in common cases presenting to the emergency department, (b) the diagnostic process (i.e., set of hypotheses, extent and quality of testing) and (c) confidence in the diagnosis. We also examine the moderating effects of case difficulty, as an earlier study indicated that DDXC may improve diagnostic performance only in more difficult cases.^21^

## 2 METHOD

### 2.1 Participants

All medical students in their fourth (out of six) academic year at the Charité – Universitätsmedizin Berlin (*N* = 300) were eligible to participate in the present study. The required sample size was estimated using G*Power for a priori power analysis.^41^ Assuming a large effect size of *d* = 0.8 for diagnostic accuracy,^42^ the total required sample size for an analysis of variance (ANOVA) with four groups (α = 0.05, power = 0.80) was 76.

### 2.2 Materials

Advanced medical students took part in the norm‐referenced computer‐based Assessing CLInical REasoning (ASCLIRE) test,^43^ where the task is to diagnose six simulated patients with dyspnea, a common symptom in the emergency department.^44^ Patients portrayed by the same male standardised actor with case‐specific prototypical symptoms and makeup were presented at the beginning of each case in 30‐s video clips showing shortage of breath.^42^, ^43^, ^45^

Per case, participants were then presented with 30 diagnostic tests on their computer screen from which they were free to choose the type, order and number of tests to administer; repeated acquisition was allowed. When clicking on a test, participants were presented with the test result in the form of a text (e.g., pulse rate), an image (e.g., ECG and chest X‐ray) or an audio clip (e.g., heart sounds and history) they had to interpret. Participants could finish data gathering at any time to provide a final diagnosis and related confidence on the next screen. They then proceeded to the next case.

**FIGURE 1:**
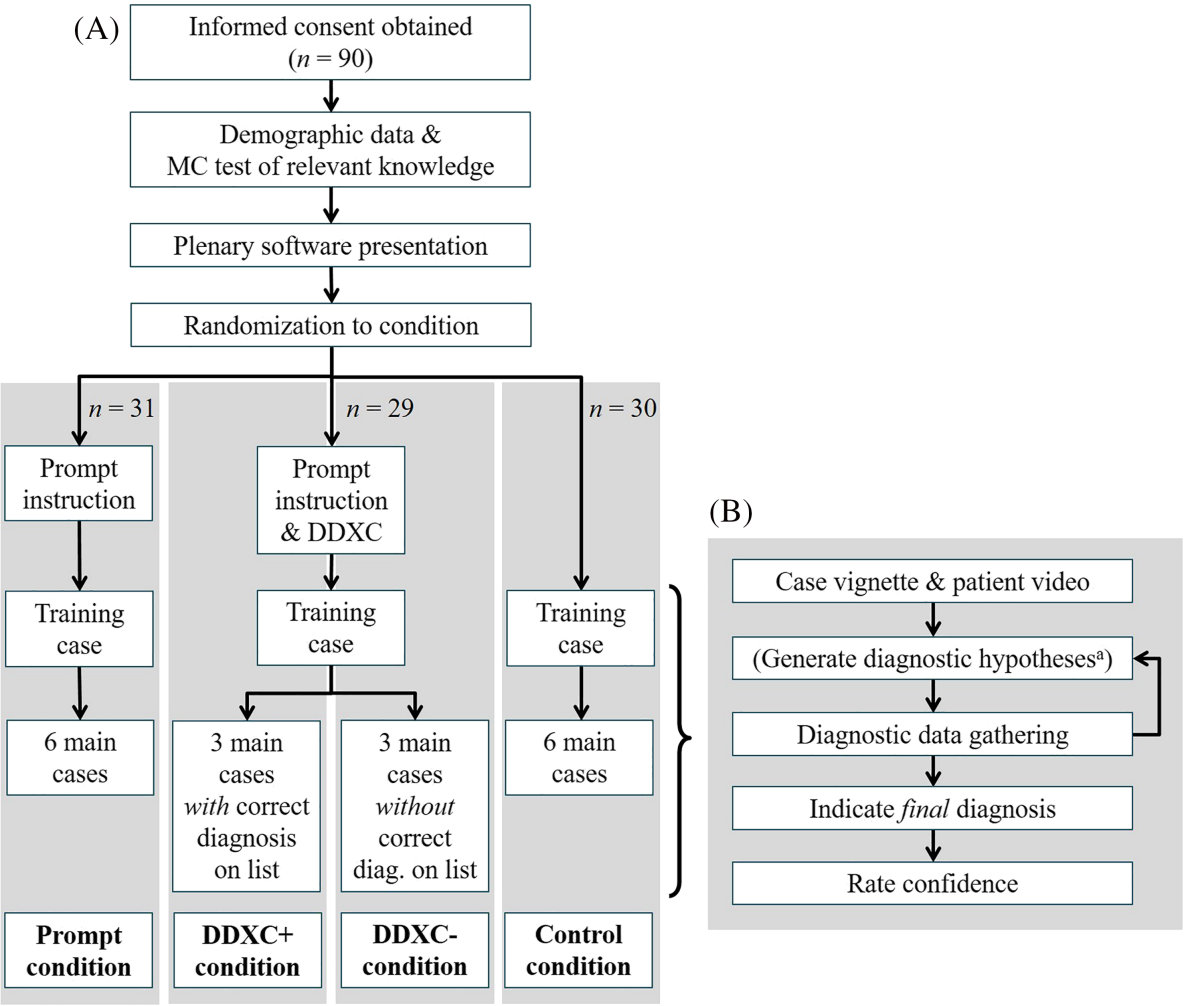
Study procedure (A) in general and (B) per (training and main) case. ^a^Only in the prompt and DDXC conditions. Note: *N* analysed = 90 [100%]. Abbreviations: DDXC, differential diagnostic checklist; DDXC−, correct diagnosis not included on list; DDXC+, correct diagnosis included on list; diag., diagnosis; MC test, multiple‐choice test

There were four study conditions (Figure 1): in the prompt condition, participants were instructed to write down all diagnostic hypotheses they considered after they knew the chief complaint and during the diagnostic process and to rank the generated diagnoses before embarking on a final diagnosis. In the two DDXC conditions, participants received additionally to the ‘prompt’ instruction a list of 17 (alphabetically ordered) diagnoses at the beginning of the experiment. Two versions of the list were designed in a counterbalanced fashion: version 1 contained the correct diagnoses for cases 1 to 3 but not for cases 4 to 6, and vice versa for version 2. Participants in the DDXC conditions were randomly assigned to receive version 1 or 2. We treated the three cases for which the checklist contained the correct diagnosis and the three cases for which the checklist did not contain the correct diagnosis as either *DDXC with correct diagnosis provided condition* (*DDXC+*) or *DDXC without correct diagnosis provided condition* (*DDXC−*), respectively. In other words, whether the DDXC contained the correct diagnosis or not varied within subject. Participants were pointed to the fact that the DDXC would not necessarily contain the correct diagnosis. They were instructed to list all generated diagnoses including diagnoses from the DDXC as well as other diagnoses they thought of. In the control condition, participants were only asked to type in their final diagnosis at the end of their information search.

### 2.3 Procedure

Upon arrival, participants read the study description and signed the consent form. After answering demographic questions, participants took a 22‐item multiple‐choice test to assess their subject‐relevant knowledge. Next, participants received a demonstration of how to work on the clinical cases. Participants were then randomised to one of the four conditions.

Participants first familiarised themselves with the interface through a practice case with the condition‐specific instructions and then worked on the six randomly ordered main cases in a computer pool while wearing headphones, without receiving any feedback on their performance. The complete session lasted about 1 h, for which participants were compensated with €20 ($22 USD at the time).

### 2.4 Ethics

Participation was voluntary and the Institutional Review Board of the Charité – Universitätsmedizin Berlin granted study approval (EA4/096/16).

### 2.5 Outcomes and measurements

#### 2.5.1 Case difficulty

Case difficulty was determined as the mean accuracy across students per case across conditions.^43^, ^45^

#### 2.5.2 Characteristics of generated lists

We recorded the number of diagnostic hypotheses considered and whether the correct diagnosis was on the self‐generated lists in the intervention conditions and, if so, ranked in which position.

#### 2.5.3 Diagnostic accuracy

Diagnostic accuracy of all final diagnoses was rated by three experts as either incorrect (0) or correct (1).^43^ Experts were board‐certified internists and emergency physicians blinded to the study condition. Inter‐rater reliability was almost perfect (Fleiss kappa = 0.841, *P* < 0.001).^46^ We used a majority rule to aggregate their ratings. In a previous study, mean accuracy ranged between 0.36 and 0.78, depending on the case.^43^

#### 2.5.4 Information acquisition measures

Similar to previous studies,^42^, ^43^ we determined three information acquisition measures: (1) the number of diagnostic tests acquired from the 30 available, (2) relevance of each diagnostic test in each case as the percentage of experts who had acquired each test before knowing the correct diagnosis in a previous study^43^ and (3) the time participants needed for diagnosing a case in the experiment (time on case).^47^

#### 2.5.5 Confidence

Participants recorded their confidence in the correctness of the diagnosis on a 10‐point ordinal scale ranging from 10 (*least confidence*) to 100 (*highest confidence*).^47^, ^48^ From this, we calculated a self‐monitoring index per person across cases to capture the extent to which confidence varied as a function of the likelihood of being correct.^49^, ^50^, ^51^ In detail, we first calculated, per person, the mean confidence for all the person's incorrect answers and the mean confidence for all the person's correct answers. We then subtracted the mean confidence for incorrect answers from the mean confidence for correct answers per person. This difference constitutes the self‐monitoring index.^47^, ^48^ Self‐monitoring indices may thus range from +90 (perfect self‐monitoring, if participants indicated the highest confidence of *M* = 100 for all their correct answers and the lowest confidence level of *M* = 10 for all their incorrect answers) to −90.

### 2.6 Statistical analyses

We used linear mixed‐effects models to analyse the impact of our interventions on diagnostic accuracy and the effect they have on the diagnostic process and confidence. We used the procedures provided in the lme4 package for fitting the linear mixed models^52^ in R.^53^

To address the research questions, we fitted nine separate models: first, three models with list characteristics as the dependent variables (list length, position of correct diagnosis on participants' lists and accuracy of listed diagnoses); second, one model including diagnostic accuracy as the dependent variable; third, three models with indicators of data‐gathering behaviour as dependent variables (number of tests, relevance of test and time on case) and, finally, two models where participants' confidence and self‐monitoring indices were entered as dependent variables.

Across all models, we included subject ID as random intercept. Furthermore, we included condition (prompt, DDXC*+*, DDXC− and control) and case difficulty (i.e., mean accuracy per case across participants) as fixed effects in all models. Where meaningful, we included the accuracy of the final diagnosis per participant as a fixed effect, too.

We used conventional thresholds for levels of statistical significance (*P* < 0.05; *P* < 0.01; *P* < 0.001) and did not adjust these thresholds for multiple comparisons because we did not consider spurious statistically significant effects to be an issue in the current application.

Raw data are available (under https://osf.io/92swt/?view_only=faec41999e4848ef9535139dfe982c1c).

## 3 RESULTS

### 3.1 Participants

Ninety students in their fourth academic year participated in the study. Random assignment to condition was effective; participant groups were similar with regard to the proportion of females, mean age, clinical semesters and task‐specific medical knowledge (Table 1).

**TABLE 1:** Participant demographics

	Prompt condition (*n* = 31)	DDXC conditions (*n* = 29)	Control condition (*n* = 30)
Gender			
Female	20 (65%)	19 (64%)	20 (67%)
Male	11 (35%)	10 (36%)	10 (33%)
Age, years, mean (SD)	23.26 (1.84)	23.34 (2.27)	24.70 (3.50)
Semesters, number, mean (SD)	7.32 (0.48)	7.28 (0.46)	7.33 (0.48)
Task‐specific medical knowledge^a^, mean (SD)	61.29 (9.38)	57.27 (10.13)	57.27 (13.38)

Abbreviations: DDXC, differential diagnosis checklist; SD = standard deviation.

^a^ Calculated from the multiple‐choice test as percentage of correctly answered items.

### 3.2 Case difficulty

**FIGURE 2:**
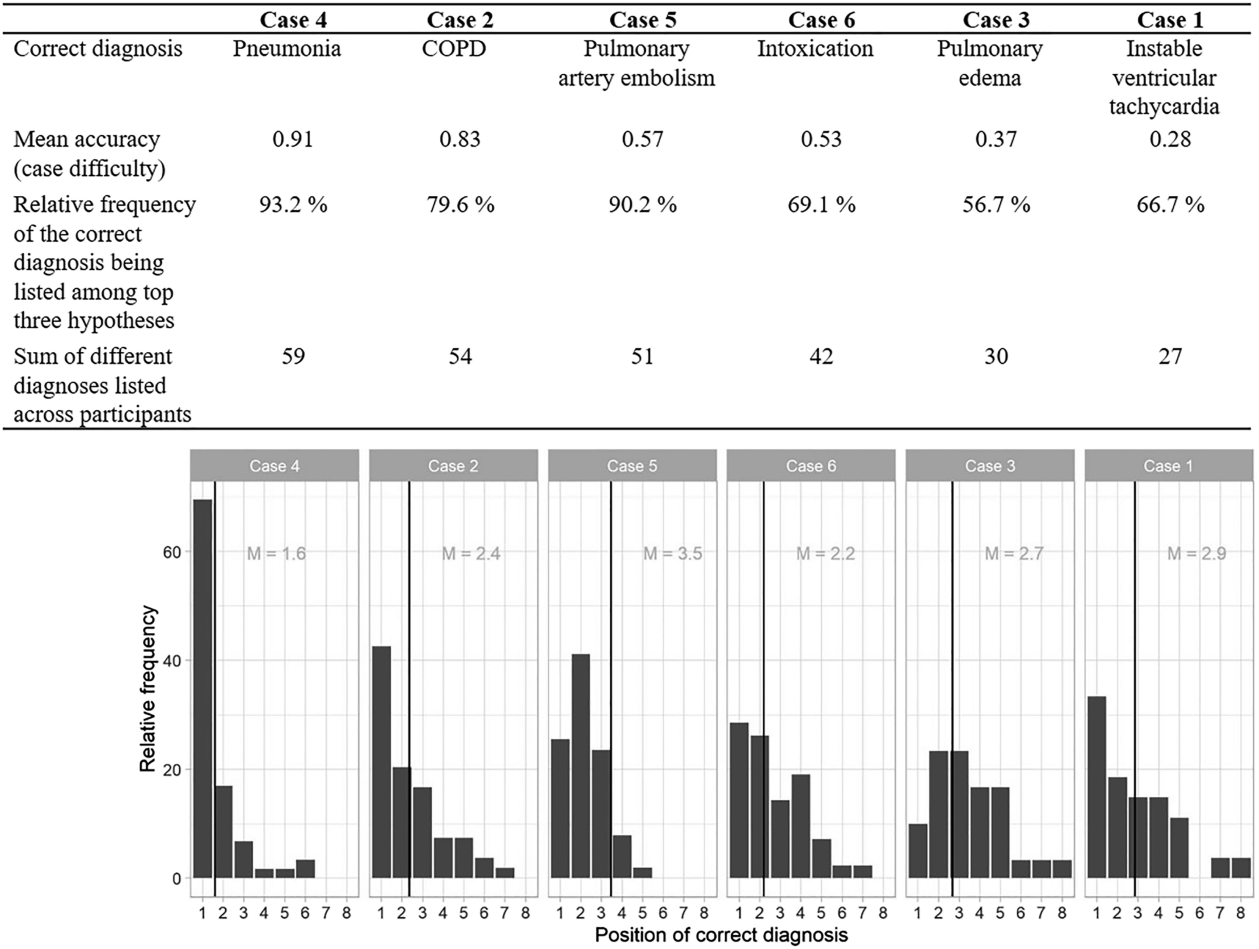
Characteristics of the six main cases, ordered by decreasing difficulty (i.e., mean accuracy): the correct diagnosis, mean accuracy and the total number of different diagnoses listed per case are noted in the table. Below, the bar chart displays the relative frequency of how often the correct diagnosis appeared on the first, second and subsequent positions on the participants' own lists, per case. The vertical lines indicate the average position of the correct diagnosis. Abbreviations: COPD, chronic obstructive pulmonary disease

Similarly to previous studies,^43^, ^45^ case difficulty was calculated as the average accuracy per case across participants; higher values thus indicate lower difficulty. Difficulty ranged from *M* = 0.28 (*SD* = 0.44) in case 1 (instable ventricular tachycardia) to *M* = 0.91 (*SD* = 0.28) in case 4 (pneumonia; Figure 2).

### 3.3 Outcome measures

In the following, we will report for each outcome variable first the descriptive results and then results from mixed‐effects models. Please refer to Table 2 for all descriptive results (column 2) and an overview of the results from mixed‐effects models (column 3) that control for case difficulty and (if appropriate) accuracy of final diagnosis. Please refer to Tables S1 to S4 for detailed statistical results.

**TABLE 2:** Descriptive (column 2) and statistical results (column 3) for all dependent variables

Variable	Observed mean [95% CI]	Estimated difference between mean and baseline [95% CI]^a^
List characteristics^b^		
Length		
Control	—	
Prompt	3.72 [3.48–3.97]	Baseline
DDXC‐	3.86 [3.52–4.20]	0.09 [−0.45–0.63]
DDXC+	3.43 [3.11–3.75]	−0.22 [−0.76–0.33]
Accuracy of listed diagnoses (0–1)		
Control	—	
Prompt	0.69 [0.63–0.76]	Baseline
DDXC‐	0.66 [0.56–0.76]	−0.01 [−0.12–0.90]
DDXC+	0.89 [0.82–0.95]	**0.16 [0.06–0.27]**
Position of correct diagnosis		
Control	—	
Prompt	2.57 [2.32–2.81]	Baseline
DDXC‐	2.42 [2.10–2.74]	−0.24 [−0.79–0.31]
DDXC+	2.04 [1.78–2.30]	**−0.55 [−1.07 – −0.03]**
Final diagnosis		
Accuracy (0–1)		
Control	0.49 [0.42–0.56]	Baseline
Prompt	0.53 [0.46–0.60]	0.04 [−0.05–0.14]
DDXC‐	0.43 [0.32–0.53]	−0.03 [−0.15–0.09]
DDXC+	0.75 [0.66–0.84]	**0.23 [0.11–0.34]**
Information gathering		
Number of tests (0–30)		
Control	24.12 [22.64–25.59]	Baseline
Prompt	22.00 [20.71–23.29]	−2.05 [−5.54–1.45]
DDXC‐	23.38 [21.44–25.32]	−0.90 [−4.61–2.81]
DDXC+	23.07 [20.99–25.15]	−0.58 [−4.31–3.15]
Time on case (m:s)		
Control	2:59 [2:44–3:14]	Baseline
Prompt	3:28 [3:11–3:44]	0.50 [−0.04–1.05]
DDXC‐	4:20 [3:47–4:52]	**1.29 [0.67–1.90]**
DDXC+	3:52 [3:25–4:19]	**1.05 [0.42–1.67]**
Relevance of tests (0–100)		
Control	58.03 [56.69–59.37]	Baseline
Prompt	60.13 [58.84–61.43]	2.05 [−1.02–5.12]
DDXC‐	60.69 [58.71–62.67]	2.55 [−0.78–5.87]
DDXC+	59.09 [57.05–61.13]	0.91 [−2.42–4.25]
Confidence		
Confidence (10–100)		
Control	59. 11 [55.70–62.52]	Baseline
Prompt	65.48 [62.16–68.81]	5.86 [−0.88–12.60]
DDXC‐	68.28 [64.06–72.49]	**10.08 [2.73–17.43]**
DDXC+	72.76 [68.72–76.80]	**10.44 [3.04–17.83]**
Self‐monitoring (delta confidence)		
Control	10.56 [3.84–17.28]	Baseline
Prompt	16.95 [11.08–22.82]	6.36 [−3.15–15.94]
DDXC‐	10.22 [3.07–17.39]	−0.33 [−10.52–9.86]
DDXC+	3.24 [−3.57–10.04]	−7.32 [−18.33–3.69]

*Note:* In all but the self‐monitoring analyses, the number of cases analysed was *n*_control_ = 180, *n*_prompt_ = 186, *n*_DDXC‐_ = 87 and *n*_DDXC+_ = 87. In the self‐monitoring analyses, the number of participants analysed was *n*_control_ = 29, *n*_prompt_ = 28, *n*_DDXC‐_ = 22 and *n*_DDXC+_ = 17. Results in bold indicate *P* < 0.05; for a complete report of the results of mixed‐effects models, see the supporting information.

Abbreviations: CI, confidence interval; m, minutes; s, seconds.

^a^ Differences between the mean of the condition and the baseline, estimated from the mixed‐effects models that control for case difficulty and, where appropriate, for accuracy of the final diagnosis; for a complete report of the results of mixed‐effects models, see the supporting information.

^b^ Note that the control group did not generate a differential diagnosis list.

#### 3.3.1 Characteristics of generated lists

Participants generated on average *M* = 3.72 (*SD* = 1.70) alternative diagnoses in the prompt condition, *M* = 3.86 (*SD* = 1.61) diagnoses when receiving a DDXC that did not include the correct diagnosis (DDXC−) and *M* = 3.43 (*SD* = 1.52) diagnoses when receiving a DDXC that included the correct diagnosis (DDXC+; Table 2). Note that the control group did not generate a differential diagnosis list. List length varied from *M* = 3.07 (*SD* = 1.47) in the easiest case 4 to *M* = 4.28 (*SD* = 1.80) in the second to hardest case 3. In correctly solved cases, list length was on average *M* = 3.48 (*SD* = 1.65), and in incorrect cases *M* = 3.95 (*SD* = 1.60). Results from mixed models indicated that list lengths did neither differ between intervention conditions, nor between difficulty levels, nor between correctly versus incorrectly solved cases (see also Table S1).

Next, we determined the accuracy of the listed diagnoses, that is, whether participants' own lists contained the correct diagnosis or not. We found that, compared to the prompt condition (*M =* 0.69, *SD* = 0.46), accuracy of the lists was higher in the DDXC*+* condition (*M* = 0.89, *SD* = 0.32) but not in the DDXC− condition (*M =* 0.66, *SD* = 0.48). The likelihood of the correct diagnosis being listed decreased from *M* = 0.98 (*SD* = 0.13) in the easiest case 4 to *M* = 0.45 (*SD* = 0.50) in the most difficult case 1. Results from mixed models indicated that the increase in list accuracy from the prompt to the DDXC*+* condition was statistically significant, *P* = 0.002 but not the decrease in the DDXC− condition. Further, the decrease in list accuracy with increasing case difficulty was statistically significant, *P* < 0.001.

As shown by Figure 2, the correct diagnostic hypothesis was ranked among the top three hypotheses in the majority of cases, namely, in 56.7% (case 3) to 93.2% (case 4). Compared to the prompt condition (*M* = 2.57, *SD* = 1.70), participants ranked the correct diagnosis earlier in the DDXC+ (*M* = 2.04, *SD* = 1.24) and in the DDXC− conditions (*M* = 2.42, *SD* = 1.52). Also, the position varied with case difficulty, though not linearly, with the correct diagnosis being ranked earliest in the easiest case 4 (*M* = 1.59, *SD* = 1.18) and latest in the second to hardest case 3 (*M* = 3.47, *SD* = 1.74), indicating some case specificity. In correctly solved cases, the correct diagnosis appeared on position *M* = 2.22 (*SD* = 1.52) and in incorrectly solved cases on position *M* = 2.89 (*SD* = 1.54). Results from mixed models indicated that the difference between control and DDXC+ was statistically significant, *P* = 0.038 but not the difference between control and DDXC−. Also, the differences in the position of the correct diagnosis between cases of different difficulty levels were statistically significant, *P* < 0.001, but not between correctly versus incorrectly solved cases.

#### 3.3.2 Did using checklists improve accuracy of the final diagnosis?

Diagnostic accuracy of the final diagnosis increased from 0.49 (*SD* = 0.50) in the control condition to 0.75 (*SD* = 0.44) in the DDXC*+* condition. Accuracy only slightly increased in the prompt condition (*M* = 0.53, *SD* = 0.50) and slightly decreased in the DDXC− condition (*M* = 0.43, *SD* = 0.50). Results from mixed models revealed a statistically significant increase in the DDXC+ condition, *P* < 0.001, and no statistically significant differences between control and DDXC− nor between control and the prompt condition (Tables 2 and S2).

#### 3.3.3 Did using checklists affect information‐gathering behaviour?

Of 30 available tests, participants acquired between *M* = 22.00 (*SD* = 8.97) in the prompt condition and *M* = 24.12 (*SD* = 10.12) tests in the control condition. The extent of information gathering ranged from *M* = 21.26 (*SD* = 8.83) tests in the easiest case 4 to *M* = 25.28 (*SD* = 12.13) tests in the intermediate case 6. More tests were administered when the final diagnosis was incorrect (*M* = 24.15, *SD* = 9.92) than when it was correct (*M* = 22.19, *SD* = 9.18). In line with this result, participants spent more time on a case when the final diagnosis was incorrect (*M* = 3:50 min, *SD* = 2:13) than correct (*M* = 3:14 min, *SD* = 1:53). Compared to the control condition (*M* = 2:59 min, *SD* = 1:44), participants took more time to make a diagnosis in the prompt condition (*M* = 3:28 min, *SD* = 1:53), in the DDXC+ condition (*M* = 3:52 min, *SD* = 2:09), and even more in the DDXC− condition (*M* = 4:20 min, *SD* = 2:36). The time spent on information gathering varied between *M* = 2:49 min (*SD* = 1:37) in the easiest case 4 and *M* = 4:16 min (*SD* = 2:19) in the second to hardest case 3. Results from mixed models revealed no statistically significant differences in the number of tests between conditions, nor between cases of different difficulty levels, but between correctly versus incorrectly solved cases, *P* = 0.040 (Table S3). They also revealed an effect of condition on the time spent on the case, *P* < 0.001 as well as of final accuracy, *P* = 0.004, but not of case difficulty.

Relevance of acquired tests was similarly high across conditions, varying between *M* = 58.03 (*SD* = 9.17) in the control condition and *M* = 60.69 (*SD* = 9.43) in the DDXC− condition. Average relevance was lowest in the easiest case 4 (*M* = 55.95, *SD* = 9.62) and highest in the intermediate case 5 (*M* = 64.84, *SD* = 6.46). Average relevance in correctly solved cases was as high (*M* = 59.48, *SD* = 9.20) as in incorrectly solved cases (*M* = 59.20, *SD* = 9.38). Results from mixed models revealed no statistically significant differences between conditions nor between correct and incorrect cases, but between cases of different levels of difficulty, *P* = 0.005, indicating some case specificity.

#### 3.3.4 Did using checklists affect confidence and self‐monitoring?

Compared to the control condition (*M* = 59.11, *SD* = 23.36), confidence was higher in the prompt condition (*M* = 65.48, *SD* = 23.11) and even higher in the DDXC− (*M* = 68.28, *SD* = 20.07) and DDXC+ conditions (*M* = 72.76, *SD* = 19.21; Table 2). Confidence was lowest in the intermediate case 6 (*M* = 56.56, *SD* = 25.93) and highest in the easiest case 4 (*M* = 72.33, *SD* = 20.23). Confidence in correct diagnoses was higher (*M* = 71.97, *SD* = 19.75) than in incorrect diagnoses (*M* = 56.93, *SD* = 23.03). We then calculated the difference in confidence between incorrect and correct answers per person, known as the self‐monitoring index (possibly ranging from −90 to +90 with positive values indicating better self‐monitoring).^47^ Mean self‐monitoring was lowest in the DDXC+ condition (*M* = 3.24, *SD* = 18.70) and highest in the prompt condition (*M* = 16.95, *SD* = 16.68). Results from mixed models indicated that the observed difference in confidence between the control and DDXC− and DDXC+ conditions were statistically significant, *P* = 0.007 and *P* = 0.006, respectively, but not the difference between control and prompt (Table S4). Differences in confidence between cases of different difficulty were not statistically significant, but between correctly and incorrectly solved cases, *P* < 0.001. There were no statistically significant differences in self‐monitoring between conditions.

## 4 DISCUSSION

Diagnostic checklists have been proposed as a handy means to reduce diagnostic errors, especially missed diagnoses.^14^, ^28^ In this experimental study, we investigated how the use of a prompt to generate and write down alternative diagnoses (prompt) and, additionally, providing a differential diagnosis checklist (DDXC) affect the diagnostic accuracy as well as information gathering and confidence. Results indicate that using a generic prompt does not improve diagnostic accuracy, which concurs with previous research.^21^, ^26^, ^30^, ^54^ Results further indicate that only providing a DDXC that included the correct diagnosis increased diagnostic accuracy, which is in line with one previous study.^21^ In fact, unlike this previous study, the benefit was not restricted to difficult cases. However, when the DDXC did not contain the correct diagnosis, accuracy tended to be lower than in the control group that did neither contain the instruction to list hypotheses nor a differential checklist, although not statistically significantly.

Moreover, we observed that no intervention had an impact on the extent nor relevance of acquired diagnostic tests compared to the control condition. The only drawback we found was that using a DDXC, irrespective of whether it contained the correct diagnosis or not, increased the time spent on the diagnostic process. Diagnostic time may be, of course, crucial in settings such as the emergency department and likely depends on the length of the provided checklist, which was quite long (17 diagnoses) in our study. It may be worthwhile to test a more dynamic version of a checklist such as an electronic diagnostic decision support tool,^55^ which updates the list of suggested alternative diagnoses with each new incoming piece of information and which may be shorter.^6^, ^7^, ^56^

Further, we found that providing participants with DDXCs (vs. not) increased their confidence in their diagnosis. This result is in line with a large body of research showing that receiving advice generally increases confidence.^57^ Practically more important than raw confidence, however, would be better self‐monitoring, that is, a larger increase of confidence in a correct diagnosis versus no change or a decrease of confidence in an incorrect diagnosis. We did not observe any difference in self‐monitoring between groups, a finding that needs to be interpreted with caution though due to only six cases per participant in our study.

Only using a prompt instead of a DDXC would arguably be the more efficient and handy (because symptom‐independent) solution to the problem of diagnostic error. Our failure to show its effectiveness might have different reasons. First, the prompt may not have been specific enough to trigger the generation of more diagnostic hypotheses. Analysing the generated lists revealed that participants indeed entertained only few (i.e., roughly 4) alternative diagnoses, which is in line with previous studies where participants did *not* receive any prompt.^9^, ^10^ However, caution is warranted as this result might have occurred due to the nature of our cases, which were constructed as rather prototypical presentations^43^ and thus may not have prompted a very large and diverse set of hypotheses.

Second, only reminding participants of one—despite crucial—step might not have been enough to improve diagnostic accuracy. Similar to other generic checklists, it might be necessary to provide a reminder of several (if not all important) steps. It might also be necessary to give more concrete guidance on *how* to perform those steps such as on *how* to generate more alternative diagnoses or on how to test those hypotheses, for example, with the help of structured reflection.^13^ Also, previous research suggests that only early considerations of alternative diagnoses improve accuracy.^7^ Thus, future studies should investigate whether variations to our prompt that specify the time (e.g., as early as possible) or number of alternative diagnoses and/or include multiple steps yield better results.

Together, our findings corroborate with research suggesting that providing content‐specific knowledge (here in the form of symptom‐related checklists) rather than general debiasing procedures^33^ may increase performance of (junior) physicians.^27^, ^32^, ^58^ At the same time, the finding that decision aids such as our DDXCs may be potentially harmful in case of being incomplete calls into question their overall benefit, given that it is impossible to list all relevant diagnoses in practice. Even more sophisticated ways of presenting diagnostic decision support, such as in the form of a computer aid, bear the potential to be incomplete or even incorrect. Therefore, more systematic research into the effect of missing and incorrect advice on diagnostic accuracy is needed.^37^, ^59^

### 4.1 Limitations

Our findings of limited benefits of checklists may call into question the suitability of checklists for the diagnostic process as a whole. However, it needs to be acknowledged that we tested only three types of interventions here. Also, our experimental setup sets limitations to the generalizability of our results to clinical practice, to different presenting complaints, to more experienced physicians and other (less ill‐defined) settings. Also, generalisability to more atypical case presentations is limited, as cases here were designed as rather unambiguous, prototypical case presentations, which are common in the emergency department. Previous studies have argued that common cases tend not to benefit from (checklist‐induced or instructed) reflection.^13^, ^21^ Thus, although some cases were difficult (i.e., solved by few), they might not have been difficult because they were uncommon or ambiguous but because of knowledge deficits at this stage in medical education. Comparability with previous vignette studies is also limited as we did not use vignettes that present all material at once^21^ but rather cases that allowed for self‐directed navigation through the diagnostic process, which has the benefit of better resembling real‐life decision making.

### 4.2 Conclusion

To conclude, we provide evidence of the potential benefit of using a symptom‐specific differential diagnosis checklist during the diagnostic process for diagnostic accuracy without altering data‐gathering behaviour. At the same time, our finding that the correct diagnosis needs to be on the checklist limits the potential benefit of this type of intervention and suggests that how to design the most effective checklists and how to integrate them in the diagnostic process are still important questions to be answered.

## CONFLICT OF INTEREST

The authors report no conflict of interest. The authors alone are responsible for the content and writing of the paper.

## ETHICAL APPROVAL

The study was approved by the Institutional Review Board at the Charité – Universitätsmedizin Berlin (EA4/096/16) and was performed in accordance with ethical standards.

## AUTHOR CONTRIBUTIONS

JEK, SKS, SCH, FS, and WEH conceptualized the study. JEK, SCH, and FS collected the data. JEK analyzed the data. JEK drafted the manuscript. SKS, SCH, FS, and WEH conducted the revision of manuscript and substantial contributions: JEK, SKS, SCH, FS, and WEH approved the final version to be published.

## Supporting information


**Table S1.** Results of the mixed effects models for outcome variables concerning list characteristics
**Table S2.** Results of the mixed effects models for accuracy of the final diagnosis
**Table S3.** Results of the mixed effects models for outcome variables concerning data‐gathering behavior
**Table S4.** Results of the mixed effects models for outcome variables concerning confidence

## Data Availability

The data that support the findings of this study are openly available in OSF (at https://osf.io/92swt/?view_only=faec41999e4848ef9535139dfe982c1c).
