# Supplementary material for: Differential diagnosis checklists reduce diagnostic error differentially: A randomised experiment
Source: Med Educ. 2021 Aug 18;55(10):1172–82. doi: 10.1111/medu.14596 (PMC9290564; doi:10.1111/medu.14596)
Supplement: Supplementary file 1 — Table S1. Results of the mixed effects models for outcome variables concerning list characteristics Table S2. Results of the mixed effects models for accuracy of the final diagnosis Table S3. Results of the mixed effects models for outcome variables concerning data‐gathering behavior Table S4. Results of the mixed effects models for outcome variables concerning confidence [file MEDU-55-1172-s001.doc]

**Supplementary Material**

**eTable 1.** Results of the mixed effects models for outcome variables concerning list characteristics

| **Predictor** | **List length** | | | **Accuracy of listed diagnoses** | | | **Position of correct diagnosis on participants’ own lists** | | |
| --- | --- | --- | --- | --- | --- | --- | --- | --- | --- |
|  | **Estimate** | **CI** | ***P*** | **Estimate** | **CI** | ***P*** | **Estimate** | **CI** | ***P*** |
| (Intercept) | 4.25 | 3.74 – 4.76 | **<0.001** | 0.22 | 0.10 – 0.34 | **<0.001** | 4.17 | 3.56 – 4.79 | **<0.001** |
| Condition [DDXC–] | 0.09 | -0.45 – 0.63 | 0.735 | -0.01 | -0.12 – 0.09 | 0.834 | -0.24 | -0.79 – 0.31 | 0.402 |
| Condition [DDXC+] | -0.22 | -0.76 – 0.33 | 0.437 | 0.16 | 0.06 – 0.27 | **0.002** | -0.55 | -1.07 – -0.03 | **0.038** |
| Accuracy of final diagnosis | -0.26 | -0.62 – 0.10 | 0.160 |  |  |  | -0.32 | -0.72 – 0.09 | 0.124 |
| Difficulty | -0.68 | -1.41 – 0.06 | 0.070 | 0.81 | 0.63 – 0.99 | **<0.001** | -2.08 | -2.82 – -1.33 | **<0.001** |
| **Random effect** | | | | | | | | | |
| σ^2^ | 2.04 | | | 0.15 | | | 1.63 | | |
| τ_00_ | 0.61  _ID_ | | | 0.00  _ID_ | | | 0.53  _ID_ | | |
| ICC | 0.23 | | | 0.02 | | | 0.24 | | |
| *N* | 60  _ID_ | | | 60  _ID_ | | | 60  _ID_ | | |
| Observations | 359 | | | 360 | | | 263 | | |
| Marginal *R*^2^ /Conditional *R*^2^ | 0.029 / 0.253 | | | 0.209 / 0.227 | | | 0.125 / 0.339 | | |

*Note*. CI, confidence interval; DDXC-, differential diagnosis checklist without correct diagnosis; DDXC+, differential diagnosis checklist containing correct diagnosis; ICC, Intraclass Correlation Coefficient; ID, participant identification number; bold indicates significant (*P* < .05) results for better readability.

**eTable 2.** Results of the mixed effects models for accuracy of the final diagnosis

| **Predictor** | **Accuracy of final diagnosis** | | | | |
| --- | --- | --- | --- | --- | --- |
|  | **Estimate** | **CI** | | | ***P*** |
| (Intercept) | -0.08 | | -0.19 – 0.04 | 0.184 | |
| Condition [prompt] | 0.04 | | -0.05 – 0.14 | 0.370 | |
| Condition [DDXC-] | -0.03 | | -0.15 – 0.09 | 0.602 | |
| Condition [DDXC+] | 0.23 | | 0.11 – 0.34 | **<0.001** | |
| Difficulty | 0.98 | | 0.81 – 1.14 | **<0.001** | |
| **Random effect** |  |  | | |  |
| σ^2^ | 0.19 | | | | |
| τ_00_ | 0.00  _ID_ | | | | |
| ICC | 0.02 | | | | |
| *N* | 90  _ID_ | | | | |
| Observations | 540 | | | | |
| Marginal *R*^2^ /Conditional *R*^2^ | 0.233 / 0.251 | | | | |

*Note*. CI, confidence interval; DDXC+, differential diagnosis checklist containing correct diagnosis; DDXC-, differential diagnosis checklist without correct diagnosis; ICC, Intraclass Correlation Coefficient; ID, participant identification number; bold indicates significant (*P* < .05) results for better readability.

**eTable 3.** Results of the mixed effects models for outcome variables concerning data-gathering behavior

| **Predictor** | **Number of tests** | | | **Time on case** | | | **Relevance of tests** | | |
| --- | --- | --- | --- | --- | --- | --- | --- | --- | --- |
|  | **Estimate** | **CI** | ***P*** | **Estimate** | **CI** | ***P*** | **Estimate** | **CI** | ***P*** |
| (Intercept) | 25.94 | 22.99 – 28.88 | **<0.001** | 3.68 | 3.12 – 4.24 | **<0.001** | 60.17 | 57.43 – 62.91 | **<0.001** |
| Condition [prompt] | -2.05 | -5.54 – 1.45 | 0.251 | 0.50 | -0.04 – 1.05 | 0.071 | 2.05 | -1.02 – 5.12 | 0.191 |
| Condition [DDXC-] | -0.90 | -4.61 – 2.81 | 0.635 | 1.29 | 0.67 – 1.90 | **<0.001** | 2.55 | -0.78 – 5.87 | 0.133 |
| Condition [DDXC+] | -0.58 | -4.31 – 3.15 | 0.760 | 1.05 | 0.42 – 1.67 | **0.001** | 0.91 | -2.42 – 4.25 | 0.591 |
| Accuracy of final diagnosis | -1.58 | -3.09 – -0.07 | **0.040** | -0.54 | -0.91 – -0.17 | **0.004** | 1.15 | -0.42 – 2.73 | 0.152 |
| Difficulty | -1.80 | -4.86 – 1.26 | 0.249 | -0.74 | -1.51 – 0.03 | 0.061 | -4.65 | -7.88 – -1.42 | **0.005** |
| **Random effect** | | | | | | | | | |
| σ^2^ | 51.71 | | | 3.33 | | | 57.69 | | |
| τ_00_ | 39.84 _ID_ | | | 0.63 _ID_ | | | 27.73 _ID_ | | |
| ICC | 0.44 | | | 0.16 | | | 0.32 | | |
| *N* | 90  _ID_ | | | 90 _ID_ | | | 90 _ID_ | | |
| Observations | 540 | | | 540 | | | 539 | | |
| Marginal *R*^2^/Conditional *R*^2^ | 0.019 / 0.446 | | | 0.083 / 0.229 | | | 0.022 / 0.340 | | |

*Note*. CI, confidence interval; DDXC+, differential diagnosis checklist containing correct diagnosis; DDXC-, differential diagnosis checklist without correct diagnosis; ICC, Intraclass Correlation Coefficient; ID, participant identification number; bold indicates significant (*P* < .05) results for better readability.

**eTable 4.** Results of the mixed effects models for outcome variables concerning confidence

| **Predictor** | **Confidence** | | | | **Self -monitoring** | | |
| --- | --- | --- | --- | --- | --- | --- | --- |
|  | **Estimate** | **CI** | ***P*** | **Estimate** | | **CI** | ***P*** |
| (Intercept) | 50.41 | 44.23 – 56.59 | **<0.001** | 10.56 | | 3.86 – 17.25 | **0.002** |
| Condition [prompt] | 5.86 | -0.88 – 12.60 | 0.088 | 6.39 | | -3.15 – 15.94 | 0.189 |
| Condition [DDXC-] | 10.08 | 2.73 – 17.43 | **0.007** | -0.33 | | -10.52 – 9.86 | 0.949 |
| Condition [DDXC+] | 10.44 | 3.04 – 17.83 | **0.006** | -7.32 | | -18.33 – 3.69 | 0.192 |
| Accuracy of final diagnosis | 11.77 | 8.08 – 15.46 | **<0.001** |  | |  |  |
| Difficulty | 5.07 | -2.51 – 12.65 | 0.190 |  | |  |  |
| **Random effect** | | | | | | | |
| σ^2^ | 318.30 | | | | 338.12 | | |
| τ_00_ | 127.01 _ID_ | | | | 0.00 _ID_ | | |
| ICC | 0.29 | | | |  | | |
| *N* | 90 _ID_ | | | | 84 _ID_ | | |
| Observations | 540 | | | | 96 | | |
| Marginal *R*^2^/Conditional *R*^2^ | 0.125 / 0.374 | | | | 0.060 / NA | | |

*Note*. CI, confidence interval; DDXC-, differential diagnosis checklist without correct diagnosis; DDXC+, differential diagnosis checklist containing correct diagnosis; ICC, Intraclass Correlation Coefficient; ID, participant identification number; bold indicates significant (*p* < 0.050) results for better readability.
